# Proteomic Analysis of Polysaccharide-Milk Protein Interactions Induced by Chitosan

**DOI:** 10.3390/molecules20057737

**Published:** 2015-04-28

**Authors:** Chun-Chi Chen, Shui-Tein Chen, Jung-Feng Hsieh

**Affiliations:** 1 Ph.D. Program in Nutrition & Food Science, Fu Jen Catholic University, Taipei 242, Taiwan; E-Mail: kennath1980@gmail.com; 2 Department of Food Science, Fu Jen Catholic University, Taipei 242, Taiwan; 3 Institute of Biological Chemistry, Academia Sinica, Taipei 115, Taiwan; E-Mail: bcchen@gate.sinica.edu.tw

**Keywords:** chitosan, milk protein, two-dimensional electrophoresis, proteomics

## Abstract

The chitosan-induced coacervation of milk proteins was investigated using a proteomic approach. The addition of 0.8% chitosan to milk caused the milk proteins to coacervate after a 1 h incubation period. Approximately 86% of the milk proteins were present in the milk pellet fraction (MPF), and the protein concentration of the milk supernatant fraction (MSF) decreased from 29.4 ± 0.2 to 4.2 ± 0.6 mg/mL. SDS-PAGE analysis showed that the total intensities of serum albumin (BSA), α_S_-casein (α_S_-CN), *β*-casein (*β*-CN), κ-casein (κ-CN) and *β*-lactoglobulin (*β-*LG) in the MSF decreased to 8.5% ± 0.2%, 0.9% ± 0.3%, 0.7% ± 0.3%, 0.5% ± 0.2% and 15.0% ± 0.5%, respectively. Two-dimensional electrophoresis analysis indicated that α_S1_-, α_S2_-, *β*- and κ-CN and a fraction of the *β*-LG and BSA were found in the MSF following incubation with 0.8% chitosan. Isothermal titration calorimetry analysis indicated that binding of chitosan to milk proteins is an exothermic reaction based on binding titration curves of milk proteins dispersions with chitosan, and the enthalpy of binding (Δ*H*) and binding constant (*Ka*) were −7.85 × 10^4^ cal/mol and 1.06 × 10^5^/mol, respectively. These results suggested that the addition of 0.8% chitosan causes milk proteins to coacervate due to polysaccharide-protein interactions.

## 1. Introduction

Polysaccharide and protein interactions play significant roles in controlling the structure and texture of food products. These interactions are dependent on polymer and solvent conditions and may lead to phases separate in nature [1]. When the complex formed improves colloidal stability, the result is a bulk of two separated phases. Some reviews have described polysaccharide molecules that may interact with proteins and adsorb to more than one colloidal particle, thereby forming bridges, aggregated structures or regions depleted of polymer and drawing protein particles closer to one another due to incompatibility [2,3]. Recently, there have been reports on the interactions between polysaccharides and food proteins, which are applicable to products for the food industries [4]. In food, chitosan is a characteristic polysaccharide used to increase food protein coacervation [3]. Milk protein can be destabilized through interaction with polysaccharides. The interaction between chitosan and casein micelles, a cationic copolymer formed of glucosamine and *N*-acetylglucosamine has recently been investigated [5].

Chitosan is a naturally non-toxic modified food-compatible polymer [6,7,8]. It is a linear poly-saccharide composed of glucosamine and *N*-acetyglucosamine, which is obtained after deacetylation of the chitin derived from the exoskeletons of arthropods and crustaceans [9]. Chitosan is a widely functional substance, and recent studies have demonstrated that chitosan has many commercial uses including as a seed treatment, biopesticide, antioxidant and antimicrobial by helping plants fight against fungal infections in agriculture [10,11]. Interactions between chitosan and milk protein are of great importance in the design of dairy formulations such as milk [2]. One previous study reported that chitosan induced destabilization and coagulation of casein micelles via chitosan-milk protein interactions [12]. 

Milk is composed of proteins, lipids, vitamins and minerals. In general, milk proteins are divided into two groups, 80% of which are caseins and 20% whey proteins, which contain the six major milk protein groups α_S1_-, α_S2_-, *β-* or κ-CN, α-lactalbumin (α-LA) and *β*-LG [13]. Milk proteins can be processed into many types of dairy products such as cheese. Traditionally, cheese was manufactured from milk using chymosin, which is commonly used to improve the properties of dairy products [14]. Recently, the use of proteomics has led to novel experimental approaches to analyze food proteins in complex mixtures using high-resolution two-dimensional gel electrophoresis (2-DE) [15,16,17]. A proteomics method has been designed to study food components such as milk proteins [18]. In this approach, the first step was to separate the proteins. Proteins are separated by isoelectric focusing according to their isoelectric point (p*I*) along a pH gradient, which is followed by sodium dodecyl sulfate-polyacrylamide gel electrophoresis (SDS-PAGE) to measure their molecular weights in the second dimension [13]. All protein spots that are resolved and detected can be qualitatively and quantitatively studied in relation to one another [19,20]. Therefore, 2-DE has been employed to separate and identify individual milk proteins of interest. In previous studies conducted by our team, we have used an enzyme to induce the polymerization of milk proteins, which was analyzed with 2-DE technology [16].

Chitosan is a food grade additive used to improve the gel properties of food products. The addition of chitosan to milk causes the milk proteins to coacervate, however, no previous research has provided a proteomics analysis of the chitosan-induced aggregation of individual milk proteins during incubation. This study added various quantities of chitosan (0, 0.2%, 0.4%, 0.6% or 0.8%) to milk samples to evaluate its properties as a coagulant in dairy food systems. In the present study, we used SDS-PAGE, 2-DE technology and isothermal titration calorimetry (ITC) to investigate the effects of chitosan on the coacervation of milk proteins. The objective of this study was to analyze the chitosan-induced coacervation of milk proteins using a proteomics approach.

## 2. Results and Discussion

### 2.1. Effects of Chitosan on the Coacervation of Milk Proteins

**Figure 1:**
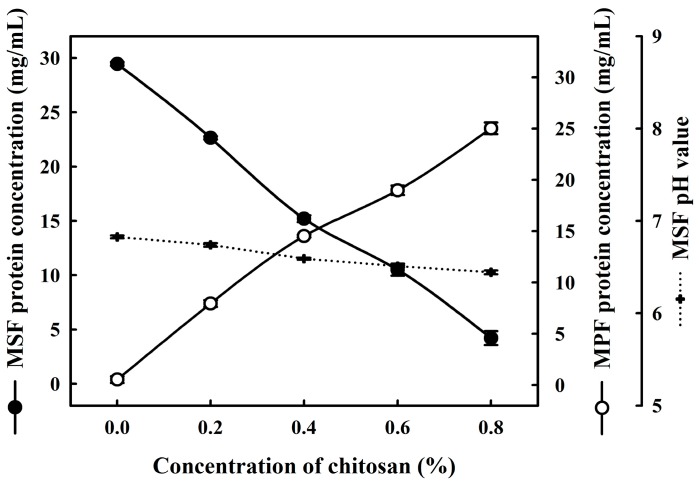
Changes in the total protein content and pH of milk with different concentrations of chitosan. ●: milk supernatant fraction, MSF; ○: milk pellet fraction, MPF.

Chitosan is a polysaccharide obtained via deacetylation of chitin consisting of *β*-(1-4)-2-acetamido-2-deoxy-*β*-d-glucopyranose and 2-amino-2-deoxy-*β*-d-glucopyranose and containing a large number of amine groups and hydroxyl groups [1]. Milk samples were incubated with different amounts of chitosan (0%, 0.2%, 0.4%, 0.6% and 0.8%) at 30 °C for 1 h, and total protein concentrations in the MSF and the MPF were and determined as shown in Figure 1. In the MSF and MPF without chitosan treatment, the total protein levels were 29.4 ± 0.2 and 0.5 ± 0.3 mg/mL, respectively, indicating that protein coacervation did not occur in milk samples without the addition of chitosan. However, the total protein in the MSF decreased with the addition of chitosan. Total protein in the MSF after the addition of chitosan decreased from 29.4 ± 0.2 mg/mL (control) to 4.2 ± 0.6 mg/mL. Otherwise, total protein in the MPF was obviously increased by the addition of 0.8% chitosan (*p* < 0.05). The pH value did not significantly change when the MSF samples were treated with different concentrations of chitosan. As previously stated, the addition of polysaccharides to milk destabilizes and coagulates casein micelles without changing the pH. Therefore, the results suggested that the addition of 0.8% chitosan causes milk proteins to coacervate due to polysaccharide-protein interactions.

Polysaccharide-protein interactions play significant roles in controlling the structure, texture, and stability of food systems. Some reports have demonstrated that various types of intermolecular forces contribute to these polysaccharide-protein interactions, including pH, covalent, ionic strength, electrostatic, excluded volume, hydrogen bonding, hydrophobic and Van der Waals interactions [21,22]. Ausar *et al.* also reported that the electrostatic and hydrophobic interactions contribute to the formation of the complex between chitosan and milk proteins [23], whereas the coacervation between chitosan and caseins seems to be determined mainly by electrostatic interactions due to chitosan being positively charged (-NH_2_^+^ groups); thus, chitosan can interact with negatively charged milk proteins (-COO^−^ groups) [24].

### 2.2. Use of SDS-PAGE to Analyze the Effects of Chitosan on Casein and Whey Proteins

**Figure 2:**
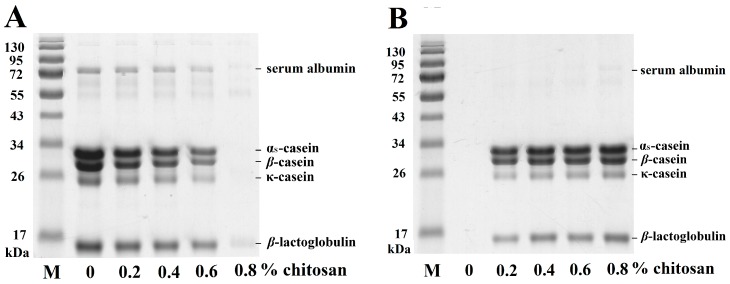
Changes in the SDS-PAGE profiles of milk samples treated with different concentrations of chitosan (0, 0.2%, 0.4%, 0.6% or 0.8%) incubated at 30 °C for 1 h. (**A**) milk supernatant fraction; (**B**) milk pellet fraction; M: protein marker.

Milk samples were incubated with different concentrations of chitosan (0%, 0.2%, 0.4%, 0.6% or 0.8%) at 30 °C for 1 h and analyzed by SDS-PAGE. Casein and whey proteins are the major milk proteins [25]. Figure 2 shows that SDS-PAGE separated the BSA, α_S_-CN, *β*-CN, κ-CN and *β*-LG proteins in the samples. Based on the SDS-PAGE results, no significant changes were observed in the milk samples without chitosan treatment. The fraction corresponding to a molecular weight of 30 kDa contained α_S_-CN, including α_S1_-CN and α_S2_-CN. The concentrations of BSA, α_S_-CN, *β*-CN, κ-CN and *β*-LG in the MSF decreased with increasing concentrations of chitosan (Figure 2A). A proportion of the α_S_-CN, *β*-CN, κ-CN, BSA and *β*-LG proteins in the MSF were depleted by 0.8% chitosan, and these proteins were apparent in the MPF (Figure 2B). Therefore, our results suggest that 0.8% chitosan could precipitate milk proteins, including BSA, CNs and *β*-CN. 

Densitograms corresponding to SDS-PAGE analysis of the milk samples treated with different concentrations of chitosan were obtained. The chitosan-milk suspension was divided into MSF and precipitate. When 0.8% chitosan was added with milk proteins, the total intensities of BSA, α_S_-CN, *β*-CN, κ-CN and *β*-LG in the MSF decreased to 8.5% ± 0.2%, 0.9% ± 0.3%, 0.7% ± 0.3%, 0.5% ± 0.2% and 15.0% ± 0.5%, respectively. Conversely, in milk precipitate, the intensity of BSA, α_S_-CN, *β*-CN, κ-CN, and a fraction of *β*-LG in the milk precipitate increased significantly following the addition of 0.8% chitosan. It has been reported that interactions between proteins and polysaccharides can induce the formation of insoluble complexes, leading to a phase separation phenomenon. The addition of polysaccharides such as chitosan to milk induces the destabilization and coagulation of casein micelles [26]; therefore, α_S_-CN, *β*-CN and κ-CN could be coacervated by chitosan [5]. In addition, *β*-LG can also be coacervated by chitosan to form *β*-LG-chitosan complexes [27]. 

### 2.3. Milk Proteins by 2-DE Analysis

**Figure 3:**
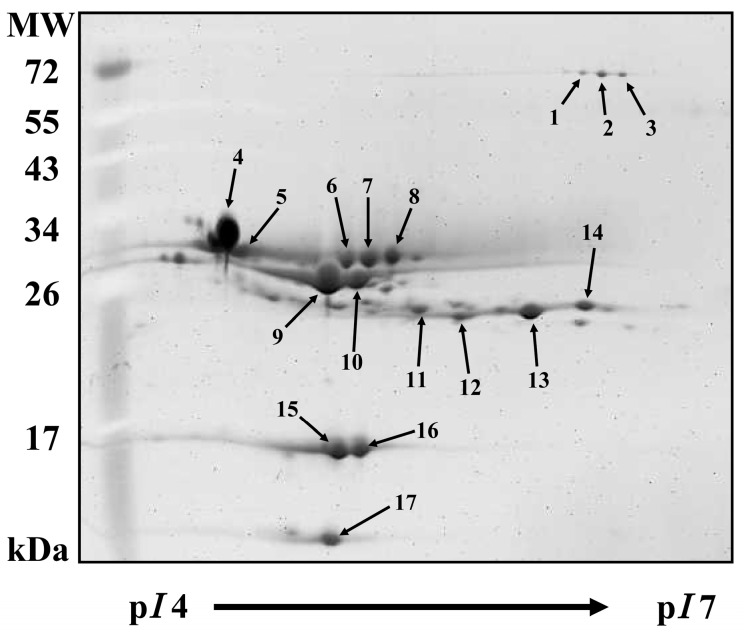
Two-dimensional polyacrylamide gel electrophoresis image of milk proteins. The sample was separated on a 12.5% SDS-PAGE gel using IPG strips (pH 4–7). The arrows indicate protein spots that were analyzed in this study. MW: molecular weight; p*I*: isoelectric point. Spots 1 to 3 were isomers of BSA, spots 4 and 5 were isomers of α_S1_-CN, spots 6 to 8 were isomers of α_S2_-CN, spots 9 and 10 were isomers of *β*-CN, spots 11 to 14 were isomers of κ-CN, spots 15 and 16 were isomers of *β*-LG, and spot 17 was α-LA.

In a previous study, Hsieh and Pan reported that these milk proteins were identified and elucidated as casein and whey proteins by direct comparison [16]. The 2-DE image of MSF without chitosan is shown in Figure 3. In total, 17 proteins were selected from the 2-DE gel and assigned individual numbers. These proteins were grouped into isomers of BSA, α_S1_-CN, α_S2_-CN, *β*-CN, κ-CN, *β*-LG, and α-LA. Spots 1 to 3 were isomers of BSA, spots 4 and 5 were isomers of α_S1_-CN, spots 6 to 8 were isomers of α_S2_-CN, spots 9 and 10 were isomers of *β*-CN, spots 11 to 14 were isomers of κ-CN, spots 15 and 16 were isomers of *β*-LG, and spot 17 was α-LA.

**Figure 4:**
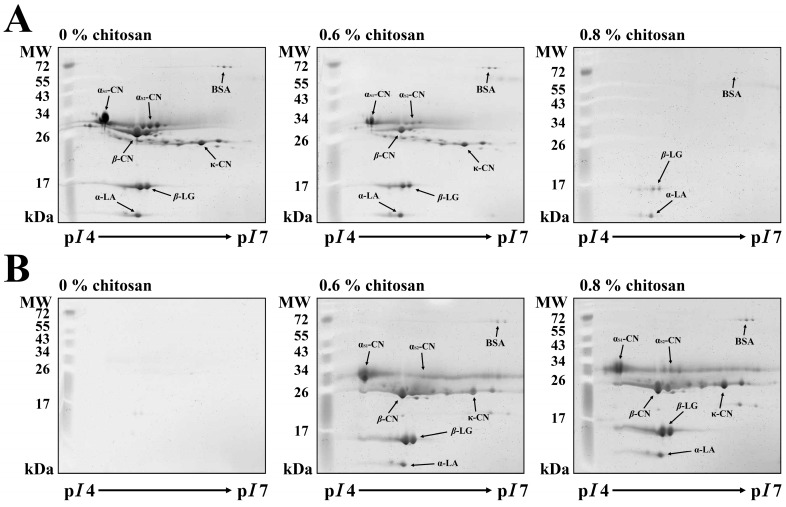
Changes in the two-dimensional polyacrylamide gel electrophoresis profiles of milk proteins following treatment with different concentrations of chitosan (0%, 0.6%, or 0.8%) at 30 °C for 1 h. (**A**) milk supernatant fraction; (**B**) milk pellet fraction; M: protein marker; MW: molecular weight; p*I*: isoelectric point.

As previously mentioned, the addition of 0.8% chitosan sufficiently coacervated the milk proteins into the milk precipitate samples. Consequently, milk samples were incubated with different amounts of chitosan (0, 0.6% or 0.8%) at 30 °C for 1 h. In 2D gels, the MSF and MPF samples were electrophoretically separated (Figure 4). After treatment with 0.8% chitosan, milk proteins in the MSF including α_S1_-CN, α_S2_-CN, *β*-CN, κ-CN and *β*-LG were depleted (Figure 4A), but they appeared in the milk precipitate (Figure 4B). Most of the whey proteins were observed in the MSF with 0.8% chitosan treatment after a 1-h incubation period, but few survived on a 2-DE gel. However, the appearance of individual α_S1_-CN, α_S2_-CN, *β*-CN, κ-CN and *β*-LG in the milk precipitate sample is evidence of the occurrence of protein coacervated by chitosan.

**Figure 5:**
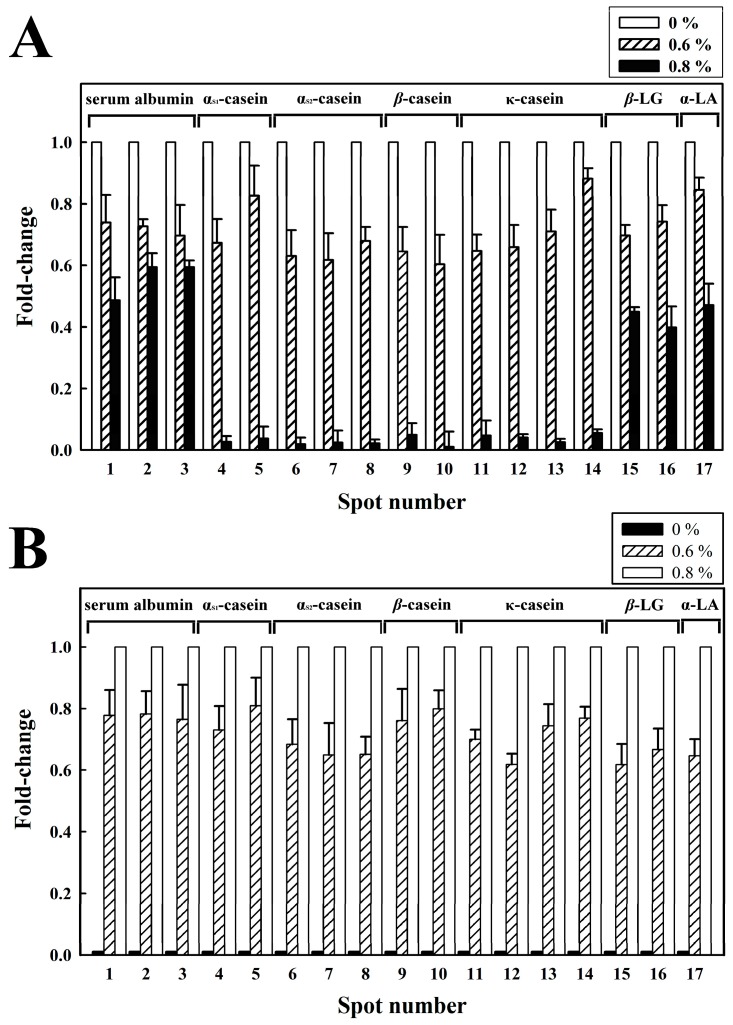
Relative abundance of milk protein spots after treatment with different concentrations of chitosan (0%, 0.6%, or 0.8%) at 30 °C for 1 h. (**A**) milk supernatant fraction; (**B**) milk pellet fraction. Histograms show the fold changes of the protein spots, which were determined using the Samespots software program.

As shown in Figure 5, densitograms corresponding to the 2-DE images of the milk samples with different amounts of chitosan (0%, 0.6% or 0.8%) were also generated, demonstrating the changes in individual milk protein levels. As previously mentioned, a portion of BSA and the majority of α_S1_-CN, α_S2_-CN, *β*-CN and κ-CN in the MSF were coacervated by the addition of 0.8% chitosan. The fold changes for BSA (spot 1), BSA (spot 2), BSA (spot 3), α_S1_-CN (spot 4), α_S1_-CN (spot 5), α_S2_-CN (spot 6), α_S2_-CN (spot 7), α_S2_-CN (spot 8), *β-*CN (spot 9), *β-*CN (spot 10), κ-CN (spot 11), κ-CN (spot 12), κ-CN (spot 13) and κ-CN (spot 14) in the chitosan-treated MSF were decreased by 0.4, 0.5, 0.5, 0.02, 0.03, 0.01, 0.02, 0.02, 0.04, 0.01, 0.04, 0.04, 0.02, and 0.05, respectively. A proportion of the *β-*LG and α-LA in the MSF were also coacervated by the addition of 0.8% chitosan. The fold changes for *β-*LG (spot 15), *β-*LG (spot 16) and α-LA (spot 17) in the chitosan-treated MSF were 0.4, 0.3 and 0.4, respectively (Figure 5A). In addition, the fold changes for α_S1_-CN, α_S2_-CN, *β-*CN, and κ-CN as well as a portion of BSA, *β-*LG and α-LA increased in the milk precipitate following the addition of 0.8% chitosan (Figure 5B). The appearance of these milk proteins in the milk precipitate demonstrates that the 0.8% chitosan treatment caused protein coacervation.

### 2.4. Microstructure of the Milk Samples with Chitosan

**Figure 6:**
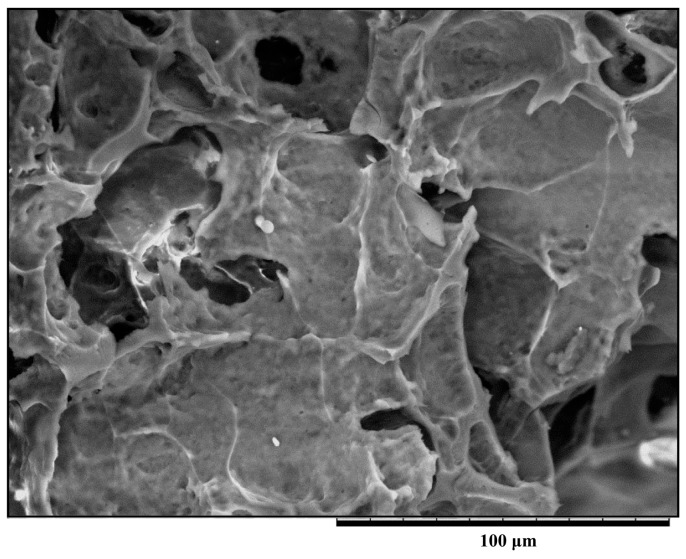
Scanning electron microscopy image of a milk MPF sample treated with 0.8% chitosan.

It has been reported that scanning electron microscopy (SEM) is a valuable technique in dairy research because it provides information on the microstructure of dairy products, which can be related to their physical properties [28]. As previously stated, protein coacervation did not occur in milk samples without added chitosan, so no chitosan-milk protein complexes formed in the milk pellet fraction (MPF). We did not observe a network of chitosan-milk protein complexes in the microstructure image of the MPF sample treated without chitosan (data not shown). In contrast, the addition of 0.8% chitosan resulted in the coacervation of milk proteins into milk precipitate to form polysaccharide-protein complexes. Figure 6 illustrates the microstructure of milk precipitate in the sample with chitosan. With a concentration of 0.8% chitosan, the micrographs showed pores sizes becoming massive and the shape of flakes becoming firm with several tiny voids. The network of the chitosan-treated milk precipitate samples was composed of fine strands in a dense arrangement. Therefore, the α_S1_-CN, α_S2_-CN, *β*-CN, κ-CN, and BSA, as well as a portion of *β*-LG were coacervated by 0.8% chitosan. These proteins must be incorporated into chitosan-treated milk precipitate samples. 

### 2.5. Milk Proteins-Chitosan Interactions Studied by ITC Measurements

The investigation of the binding energetics between chitosan and the proteins was performed via ITC measurements. The interaction of chitosan 0.8% with 5 mM phosphate buffer and pH 6.8 was characterized using ITC at 30 °C. Table 1 showed the experimental data for the titration of milk proteins with chitosan. The Δ*H* and *Ka* value of chitosan were −7.85 × 10^4^ cal/mol and 1.06 × 10^5^/mol, respectively. These indicated that milk proteins can bind with chitosan in an exothermic reaction (Δ*H* < 0). Therefore, the results suggested that the addition of 0.8% chitosan causes milk proteins to coacervate due to polysaccharide-protein interactions.

**Table 1:** Study on the interactions between chitosan and milk proteins using isothermal titration calorimetry at 30 °C.

Compound	Structure	Δ*H* (cal/mol)	*Ka*
Chitosan	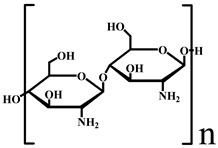	−7.85 × 10^4^	1.06 × 10^5^

The enthalpy of binding (Δ*H*) is expressed with the unit of kcal/mol. Binding constant (*Ka*) is expressed with the unit of M^−1^.

## 3. Experimental Section

### 3.1. Preparation of Milk Samples

Fresh raw milk from a healthy Holstein-Friesian cow was obtained from a local farm in Taipei, located in northern Taiwan. The milk was skimmed at 5000× *g* for 20 min, and the skim milk (29.8 mg/mL) was collected and stored at 4 °C. Chitosan oligosaccharide lactate (Lot MKBB2183 V) was purchased and obtained from Sigma Chemical Co. (St Louis, MO, USA). To investigate the effects of chitosan on the coacervation of milk proteins, milk samples with varying amounts of chitosan (0%, 0.2%, 0.4%, 0.6% or 0.8%) were incubated at 30 °C for 1 h. After incubation, the milk samples were fractionated into the milk supernatant fraction (MSF) and the milk pellet fraction (MPF) by centrifugation for 20 min (5000× *g*). The MSF samples (1 mL) were collected, and the MPF samples were re-dissolved in an equal volume (1 mL) of lysis solution containing 7 M urea, 2 M thiourea and 4% 3-[(3-cholamidopropyl)-dimethylammonio]-1-propanesulfonate prior to use.

### 3.2. Determination of Protein Concentration

The protein concentrations of the milk samples were determined using the Bio-Rad protein assay (Bio-Rad, Hercules, CA, USA). The Bio-Rad protein assay dye was diluted with four volumes of water and then mixed with the standards or milk samples. Absorbance at 595 nm was measured using a VersaMax^TM^ microplate reader (Molecular Devices Corporation, Sunnyvale, CA, USA), and bovine serum albumin (Sigma) was used as a standard.

### 3.3. SDS-PAGE Analysis

Milk samples with or without chitosan were analyzed by SDS-PAGE, according to and based on Hsieh and Chen [29]. SDS-PAGE analysis of milk samples were performed using a 12.5% separating gel and a 5% stacking gel. Each sample (0.1 mL) was mixed with sample buffer (0.9 mL, 2% SDS, 5% *β-*mercaptoethanol, 10% glycerol, 0.02% bromophenol blue, and 70 mM Tris-HCl, pH 6.8) and heated to 95 °C for 5 min. The protein ladder and samples were loaded into separate wells (7 μL/well). After electrophoresis, the gels were stained with Coomassie Brilliant Blue R-250. The stained gels were imaged using an EPSON perfection 1270 image scanner (Epson America Inc., Long Beach, CA, USA) and Gel-Pro Analyzer (version 4.0, Media Cybernetics, Inc., Bethesda, MD, USA) software programs. Changes in the electrophoretic profiles were used to evaluate protein coacervation induced by chitosan.

### 3.4. Two-Dimensional Polyacrylamide Gel Electrophoresis (2D-PAGE)

After using dimension sodium dodecyl sulfate polyacrylamide gel electrophoresis (SDS-PAGE) separation by molecular size, milk samples were analyzed by 2-DE, based on the method of Hsieh and Pan [16]. For the first step of the separation, total milk protein (100 μg) was immobilized and loaded onto pH gradient (IPG) gel strips (pH 4–7, 18 cm; GE Healthcare, Little Chalfont, United Kingdom) that had been rehydrated for 12 h in a solution containing 7 M urea, 2 M thiourea, 4% 3-[(3-cholamidopropyl)-dimethylammonio]-1-propanesulfonate, 40 mM Tris-base, 2% IPG ampholyte, 65 mM 1,4-dithioerythritol (DTE), and 0.0002% bromophenol blue. Isoelectric focusing of the strips was performed using the IPGphor 3 IEF system (GE Healthcare) at 20 °C and 6000 V for a total of 60 kVh. The strips were equilibrated for 15 min in equilibration solution (50 mM Tris-HCl, pH 8.8, 6 M urea, 2% SDS, 30% glycerol, and 2% DTE) and added to the top of a vertical 12.5% SDS-PAGE gel with 0.5% agarose. The second electrophoresis step was performed using a Protean II xi Cell system (Bio-Rad) at 10 mA per gel for 1 h followed by 45 mA per gel for 5 h until the bromophenol blue reached the bottom of the gel. After electrophoresis, the gels were immersed in 10% methanol and 7% acetic acid for 30 min and were stained overnight in 350 mL of Sypro^®^ Ruby protein gel stain solution [30]. The developed gels were digitally scanned as 2-D images using a Typhoon 9200 imaging system (Amersham Pharmacia Biotech, Piscataway, NJ, USA) and analyzed using the Samespots software program (TotalLab Inc., Newcastle-upon-Tyne, UK).

### 3.5. Microstructure Analysis

A scanning electron microscope (TM-3000, Tabletop Microscope, Hitachi, Tokyo, Japan) was used to examine the microstructure of the MPF sample in accordance with the method proposed by Lee and Kuo [31]. Briefly, the MPF samples were freeze-dried before being mounted on a bronze stub to be sputter-coated with gold using a VPS-020 Quick Coater (ULVAC Inc., Miyazaki, Japan). The samples were then examined using the scanning electron microscope at an acceleration voltage of 15 kV.

### 3.6. Milk Protein-Chitosan Interactions Studied by ITC Measurements

An isothermal titration calorimeter (ITC) was used to measure the enthalpy changes and binding affinity resulting from milk proteins-chitosan interactions. The ITC experiments were performed using an isothermal titration calorimeter (MicroCal iTC200, GE Healthcare Life Sciences, Little Chalfont, UK). Using the same buffer solution (5 mM phosphate buffer, pH 6.8), 2 μL of 0.8% chitosan with phosphate buffer was injected from the computer-controlled 39 μL microsyringe at an interval of 2 min into 280 μL of a milk, casein or whey protein solution (4 mg/mL), respectively. The temperature of the solution in the titration cell was 30 ± 0.1 °C, and the solution was stirred at 1000 rpm throughout the experiments. Measurements were performed in triplicate, and the results are reported as the mean. Control experiments were performed by making identical injections of chitosan into a cell containing buffer without milk proteins. The data were collected and analyzed using Origin Version 5.0 data analysis software (Microcal Inc.). The binding isotherms match the one-site binding model, giving values for the enthalpy of binding (Δ*H*), and the binding constant (*Ka*).

### 3.7. Statistical Analysis

Data were expressed as the means ± standard deviations. The data were analyzed using the Statistical Package for the Social Sciences software (SPSS for Windows, version 10.0.7C, SPSS Inc., Chicago, IL, USA). The statistical significance between treatments was determined by one-way ANOVA followed by Duncan’s multiple range test. Three determinations for each treatment were performed, and the significance level was set at *p* < 0.05.

## 4. Conclusions

A proteomic analysis of the effects of chitosan on the coacervation of individual milk proteins was performed. The results suggest that the addition of chitosan to milk induces the destabilization and coagulation of casein micelles. These casein micelles contain α_s1_-CN, α_s2_-CN, *β-*CN and κ-CN. Furthermore, SDS-PAGE, 2-DE and ITC showed that α_s1_-CN, α_s2_-CN, *β-*CN, and κ-CN as well as a portion of BSA, *β-*LG and α-LA were coacervated by 0.8% chitosan treatment. Our results suggest that a proteomic approach can be successfully used to analyze the effects of chitosan on individual milk proteins.
